# Sensitivity Enhancement of Modified D-Shaped Microchannel PCF-Based Surface Plasmon Resonance Sensor

**DOI:** 10.3390/s20216049

**Published:** 2020-10-24

**Authors:** Abdullah Al Noman, Emranul Haque, Md. Anwar Hossain, Nguyen Hoang Hai, Yoshinori Namihira, Feroz Ahmed

**Affiliations:** 1 Department of Electrical and Electronic Engineering, Independent University Bangaldesh, Dhaka 1229, Bangladesh; emran1612@iub.edu.bd (E.H.); fahmed@iub.edu.bd (F.A.); 2 Department of Electrical and Electronic Engineering, Bangladesh University of Business and Technology (BUBT), Dhaka 1216, Bangladesh; anwar.ee113@gmail.com; 3 School of Electronics and Telecommunication, Hanoi University of Science and Technology, Ha Noi 10000, Vietnam; hai.nguyenhoang@hust.edu.vn; 4 Faculty of Engineering, University of the Ryukyus, Okinawa 903-0213, Japan; ynamihir@tec.u-ryukyu.ac.jp

**Keywords:** surface plasmon resonance, D-Shaped photonic crystal fiber, bimetallic coating, refractive index

## Abstract

In this work, a highly sensitive dual-core configured microchannel-based plasmonic refractive index (RI) sensor was investigated, which can be used for low RI detection. Both the sensing layer and the plasmonic material layer were built outside of the fiber design to detect the surrounding medium’s RI changes. Additionally, the effects of different plasmonic materials gold (Au), silver (Ag), and copper (Cu) toward sensitivity were investigated for the same structure. An adhesive agent was used in this work, titanium dioxide (TiO_2_), and was coated on top of the plasmonic material to prevent the oxidation of Ag and Cu. The coupling strength between the fundamental mode and the surface plasmon polariton (SPP) mode was observed to be very strong due to the TiO_2_ adhesive agent. With a resolution of 7.41 × 10^−7^ RIU, maximum wavelength sensitivity (WS) of 135,000 nm/RIU and amplitude sensitivity (AS) of 3239 RIU^−1^ were achieved using the proposed sensor while using Au as a plasmonic material for an analyte RI range of 1.29–1.39. A detailed study of relevant literature revealed that the achieved wavelength sensitivity for plasmonic material gold (Au) is the highest among reported photonic crystal fiber (PCF)-surface plasmon resonance (SPR) sensors to date.

## 1. Introduction

A surface plasmon is an electromagnetic wave that propagates between the surface of a thin metal layer and dielectric medium. Photonic crystal fiber (PCF) based surface plasmon resonance (SPR) sensors exploit the plasmonic surface waves, which are the collective oscillating charge at the interface between the metal film and dielectric medium for analyte detection. By forming into the traveling wave, these waves can be driven to the interface known as surface plasmon polariton (SPP) [1]. SPPs are produced between metal–dielectric medium by coupling the electromagnetic field into intensely charged particles of plasmonic materials such as Au, Ag, Cu, Al, etc. When the wave vector of the incident light is equal to the wave vector of surface plasmonic waves, resonance occurs—this condition is also known as the phase-matching condition [2]. At the phase-matching condition, a strong coupling is established between the fundamental mode and the plasmonic mode, which results in the transfer of maximum light energy from core mode to SPP mode, and a peak loss appears. The peak loss wavelength is shifted or changed when the refractive index of the surrounding medium changes. PCF-based SPR sensors can detect the shifting of the wavelength by the property of phase-matching condition between the core guided mode and SPP mode and hence, senses the change in refractive index. These sensors are recommended to handle various biological samples like bio-sensing, mineral water examining, professional medical diagnostics, maintaining high-quality food, disease discovery, bio-imaging, real-time supervising, checking sugar and carbohydrates, environmental supervising, etc. [2,3]. SPR optical sensors are more convenient compared to Kretschmann Raether prism geometry-based sensors platforms due to the features of ease of fabrication, reusable sensor chips, low cost, and miniaturized application. 

The primary ground for building this PCF-SPR sensor is usually to determine productive coupling concerning core mode in addition to the plasmonic mode by means of optimizing the structural design of the sensor. Most of these can be achieved by building this PCF design effectively where the selection of metal layer, the diameter of air holes, depth of metal layer engage in vital purpose when it comes to greater sensitivity. [4,5]. The design having metal-coated analyte slots positioning in the cladding area was theoretically analyzed as to where gold could be used as plasmonic material. This kind of sensor demonstrated a remarkable result of higher sensitivity towards realistic applications. [6]. However, this kind of set-up of associating the plasmonic material layer towards the precise air hole as well as filling up this with the analyte is extremely difficult and challenging to implement it practically [7]. To attenuate the particular difficulty, a D-shaped PCF sensor framework is presented in which plasmonic substance will be sprayed around the flat work surface of the fiber, in addition, analyte could be sensed externally where sensitivity is 2900 nm/RIU [7]. The level of sensitivity continues to be improved considerably simply by adjusting the particular D-Shaped composition having an external analyte route covering together with gold by increasing the resonance result together with the evanescent field producing a couple of passageways. This was suggested for a broad range of analyte refractive index (RI) recognition that ranges from 1.18 to 1.36, where sensitivity is 20,000 nm/RIU [3].

The selection of plasmonic material for the sensing behavior of the PCF-SPR sensor is an important issue [8,9]. Gold is popularly chosen as a plasmonic material as it is less affected by the surrounding environment like aqueous materials that have large shifts in their resonance peaks; however, it is lossy [10,11]. Silver can be an alternative as it shows a sharper resonance peak compared to others, but it is oxidized easily by the surrounded aqueous environment [4]. A graphene layer coating with silver film can overcome the oxidation problem, but this combination is not appropriate for long-term applications [11,12]. Copper and aluminum can be opted as cheaper than gold and silver, but they are also oxidized easily. Cu film was coated with a graphene layer to overcome the oxidation problem and increase sensing performance for the π–π stacking [13,14,15]. Sensing performance has been improved significantly by applying a Titanium dioxide (TiO_2_) layer coated in between the gold film and silica glass of microchannel. In this PCF-SPR sensor, the maximum wavelength sensitivity (WS) of 51,000 nm/RIU was obtained [16]. The effects of plasmonic materials and layers on the PCF-SPR sensor were previously numerically investigated and analyzed for graphene on Ag and very few Gold layers on Ag [11]. The numerical comparison of Gold and Silver on the same PCF structure has also been studied, where silver was found to be more suitable for that specific structure [17]. Recently, the effect of various metal layers (Au, Ag–Au, Ag–graphene) has been analyzed and compared with the same PCF structure, where maximum sensitivity of 4000 nm/RIU was obtained for graphene covered silver plasmonic layer [18].

A modified D-shaped microchannel PCF outward detection-based dual-core plasmonic sensor is offered in this work where sensitivity was increased by making use of diverse bimetallic plasmonic substance layers. The detection layer and the substance layer were set on the top of the two leaky channels. The TiO_2_ layer was used to cover plasmonic material and was placed between the plasmonic material and the analyte channel. In addition to performing as a colloidal agent, TiO_2_ can improve the coupling outcomes between the fundamental core guided mode and SPP mode [16]. The improvement of the evanescent field enhanced the resonance of the proposed sensor. The evanescent field improvement was done by the two leaky channels and bimetallic microchannel. Finally, the effects on the sensing performance of the bimetallic plasmonic layers using different metals (Au–TiO_2_, Ag–TiO_2_, and Cu–TiO_2_) were analyzed and compared with the same PCF structure. To our best knowledge, no previous research has been done where those bimetallic plasmonic layers have been studied and compared using the same structure.

## 2. Numerical Design and Modeling

**Figure 1:**
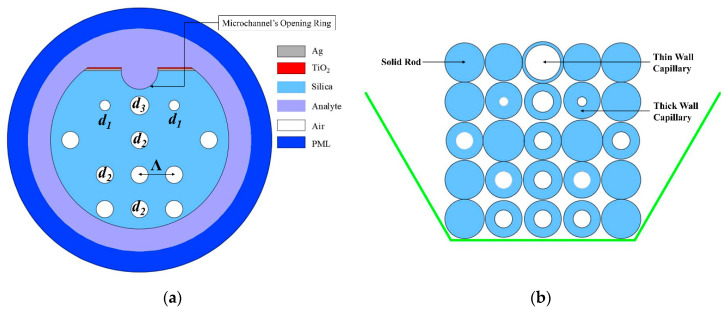
(**a**) Proposed sensor’s schematic diagram; (**b**) proposed fiber’s stacked preform.

The cross-section of the PCF-SPR sensor proposed in this work is demonstrated in Figure 1a. This particular sensor had a square lattice setting possessing a couple of tiers of air hole ring. The coupling between the core guided mode and the SPP mode was enhanced by making use of the scaling down technique towards the two air holes (d_1_) from the first ring. From the middle of the 1st ring, opposite two air holes were removed to create a dual-core configuration. This particular sensor’s probable approach of fabrication is shown in Figure 1b. In order to fabricate the fiber, the stack and drawn technique can be used where all of the solid rods as well as capillaries are pulled at a certain rate just after stacking them with each other [3,19,20]. All the tiny, big, and no air holes can be constructed using thin and thick capillaries, respectively [20]. Immediately after finishing the fabrication, the polishing process can be utilized to style the D-shaped design as well as to produce microchannel [19,21]. In this case, the chemical deposition method was applied to coat TiO_2_ and plasmonic material on the glassy surface [3,16,22].

In this case, a highly advanced electromagnetic simulation software named COMSOL Multiphysics (Version 5.4) has been used to simulate and analyze the proposed PCF-SPR sensor. To soak up the radiation power, a perfectly matched layer (PML) was incorporated into the outermost tier of the fiber. When the simulation was performed, the physics-controlled mesh was considered excellent and capable of achieving the maximum simulation accuracy. The particular parameters optimized regarding this distinct design were as follows: Λ = 3.30 µm (pitch), d_3_ = 1.80 µm, d_1_ = 1.00 µm, d_2_ = 1.65 µm, thickness of plasmonic materials (Au, Ag and Cu) and TiO_2_ are 65 and 10 nm, respectively. The microchannel’s opening was 1.75 µm, and from the Drude model, which is described in [23], the dielectric constant of silver was obtained. Similarly, refractive indexes were calculated for gold and copper, respectively, following the guidance of [24]. The Sellmeier equation was used to calculate the RI of SiO_2_, which was used as background material [25].

(1) $$n_{SiO_{2}}^{2}(\lambda )=1+\frac{B_{1}\lambda^{2}}{\lambda^{2}-C_{1}}+\frac{B_{2}\lambda^{2}}{\lambda^{2}-C_{2}}+\frac{B_{3}\lambda^{2}}{\lambda^{2}-C_{3}}$$

where the refractive index of silica is indicated by *n*_*SiO*_2__. *B_i_* and *C_i_* [*i* = 1, 2, 3] denote the Sellmeier coefficients.

The equation mentioned below is used to calculate the RI of TiO_2_ [19].

(2) $$n_{TiO_{2}}^{2}=5.913+\frac{2.441\times 10^{7}}{\left(\lambda^{2}-0.803\times 10^{7}\right)}$$

where the operating wavelength is represented by *λ* in µm.

**Figure 2:**
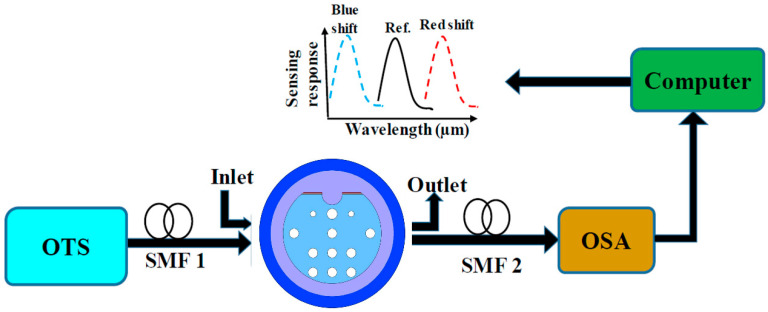
Proposed sensor’s experimental setup.

The performances of the proposed sensor were analyzed numerically. The experimental setup for the proposed sensor is illustrated in Figure 2, where the setup is incorporated with the optical spectrum analyzer (OSA), polarization controller, and optical tunable source (OTS); these components were connected in series with a Single-Mode Fiber (SMF). The D-shaped structure was used in the proposed sensor to place the sensing channel or analyte (for the target material’s RI to be sensed) at the external side. A pump was used to control the flow of analyte through the inlet–outlet channel. Redshifting (shifting towards higher wavelengths) or blueshifting (shifting towards lower wavelengths) was observed for the proposed sensor when the communal interaction between legends and analyte establishes and the shiftings were monitored by OSA.

## 3. Result and Discussion

**Figure 3:**
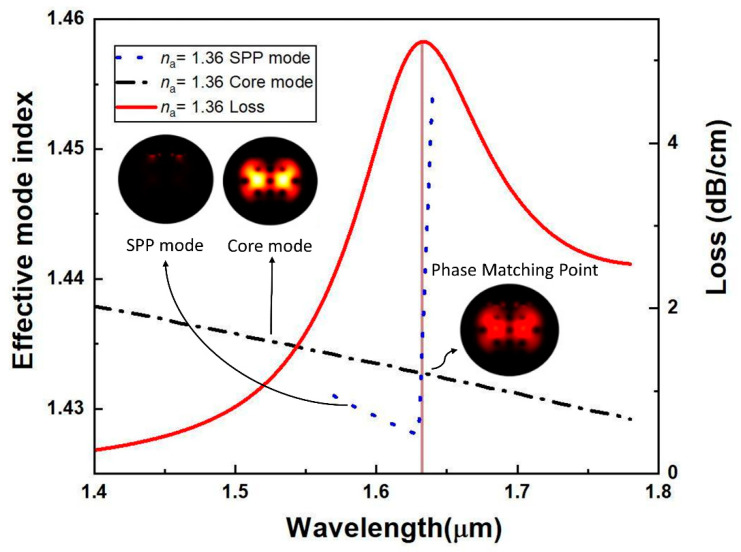
Field distribution of *n_a_* 1.36 with the surface plasmon polariton (SPP) mode, the fundamental core mode, resonance coupling, and dispersion relation for plasmon polariton mode and fundamental core mode.

For the analyte with an RI of 1.36, the field distribution of the fundamental mode and SPP mode is shown in Figure 3. In this case, the SPP mode shows up at the sensing medium and microchannel coated with plasmonic-material (Au), while the whole optical field gets trapped in the core at the fundamental mode. Figure 3 illustrates the optical field distribution at the phase-matching condition. At the resonance condition, it is observed that a strong coupling was established between the core mode and plasmonic mode, ensuring that the maximum amount of energy was delivered from the core to the SPP mode. Loss spectra and the relation of dispersion of the core mode and SPP mode are presented in Figure 3. The intersection of refractive indexes of core mode and the 2nd-order plasmonic mode was observed to have an operating wavelength of 1.63 µm, whereas the analyte had an RI of 1.36. A noticeable evanescent field was produced due to the maximum number of free electrons from the y-polarized transverse electric (TE) mode TEy rather than from the x-polarized TEx mode [16]. The phase-matching condition was met at the resonance point, which led to the transition of all the power from core mode to SPP mode. As a result, a peak loss appeared at the point where refractive indexes of the two modes intersect each other.

The propagation loss is stated as [16,19]

(3) $$\alpha =\frac{40\pi Im(n_{eff})}{In(10)\lambda }\approx 8.686\times k_{0}Im(n_{eff})\times 10^{4}\;\mathrm{dB}/\mathrm{cm}$$

here *k*_0_ = 2*π*/*λ* represents the free space wavenumber and *Im*(*n_eff_*) being the imaginary part of effective RI.

**Figure 4:**
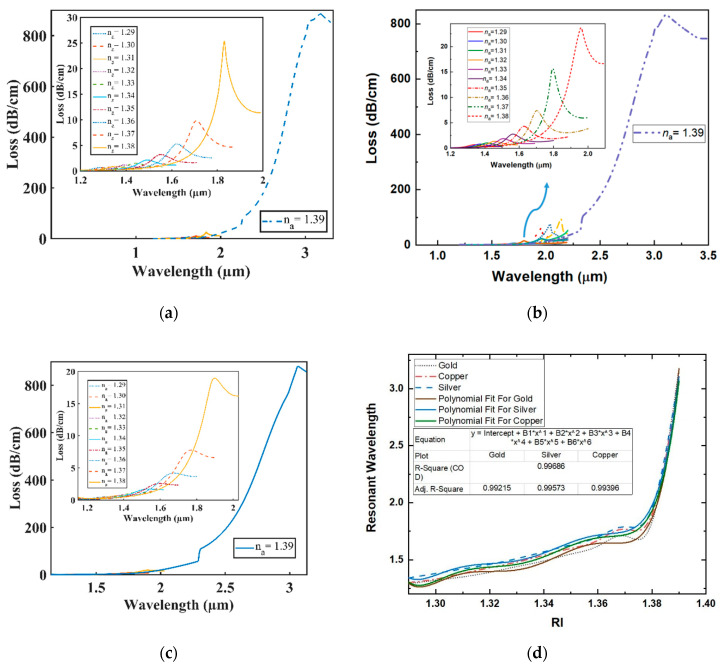
(**a**–**c**) Loss spectra with varying analyte refractive index (RI) from 1.29 to 1.39 of Au, Ag and Cu, respectively; (**d**) polynomial fit of resonant wavelengths.

Figure 4a–c illustrates the loss spectra of the resonant wavelength where the value of analyte RI ranges from 1.29 to 1.39 in the case of plasmonic material Gold, Silver, and Copper. For the proposed sensor, redshifting with the confinement loss increasing was seen each time for all the plasmonic substances due to the incomplete coupling property explained in [22]. At RI = 1.39, when the wave vector of the transmitted light matches the surface plasmonic wave vector, the highest light penetration to the metal occurs for each plasmonic material, which creates a significantly strong coupling between the core mode and SPP mode. As a result, the highest confinement loss was observed as well as significant redshifting of peak loss wavelength for the proposed sensor at RI = 1.39. The polynomial fit R–square was 0.992, 0.995, and 0.993 for Gold, Silver and Copper, respectively (see Figure 4d), which indicates a well-fitting agreement.

The wavelength sensitivity and resolution of the proposed sensor can be calculated from Equations (4) and (5) [19,26], respectively. The proposed sensor shows a maximum WS of 1,35,000, 116,000, and 117,000 nm/RIU and a resolution of 7.41 × 10^−7^, 8.62 × 10^−7^, and 8.54 × 10^−7^ RIU when peak loss shifting was observed for *n_a_* = 1.38 to *n_a_* = 1.39 for plasmonic material Gold, Silver, and Copper, respectively. The analyte’s RI range varied from 1.29 to 1.39 for all plasmonic materials with a 0.01 step size. The proposed sensor showed a minimum loss of 0.65, 0.74, and 0.48 dB/cm at 1290, 1340 and 1300 nm resonance wavelengths and a maximum loss of 885, 829, and 879 dB/cm at 3180, 3110, and 3070 nm resonance wavelengths for Au, Ag and Cu, respectively. To establish the strong coupling between the core mode and plasmonic mode, massive propagation loss was found at *n_a_* = 1.39. Furthermore, from [27], the figure of merit (FOM) (Figure of Merit = Sensitivity/FWHM) was calculated., and the maximum FOM achieved was 3375, 2320, and 3900 for Gold, Silver and Copper, respectively, for the analyte RI of 1.38.

(4) $$WS\;[nm/RIU]=\frac{\Delta \lambda_{peak}}{\Delta n_{a}}$$

where Δ*λ_peak_* is the peak wavelength shift at resonance point and Δ*n_a_* denotes the difference of analyte RI.

(5) $$R=\Delta n_{a}\Delta \lambda_{min}/\Delta \lambda_{peak}RIU$$

where Δ*λ_peak_* is the peak wavelength and Δ*λ_min_* is the lowest resolution of wavelength.

**Figure 5:**
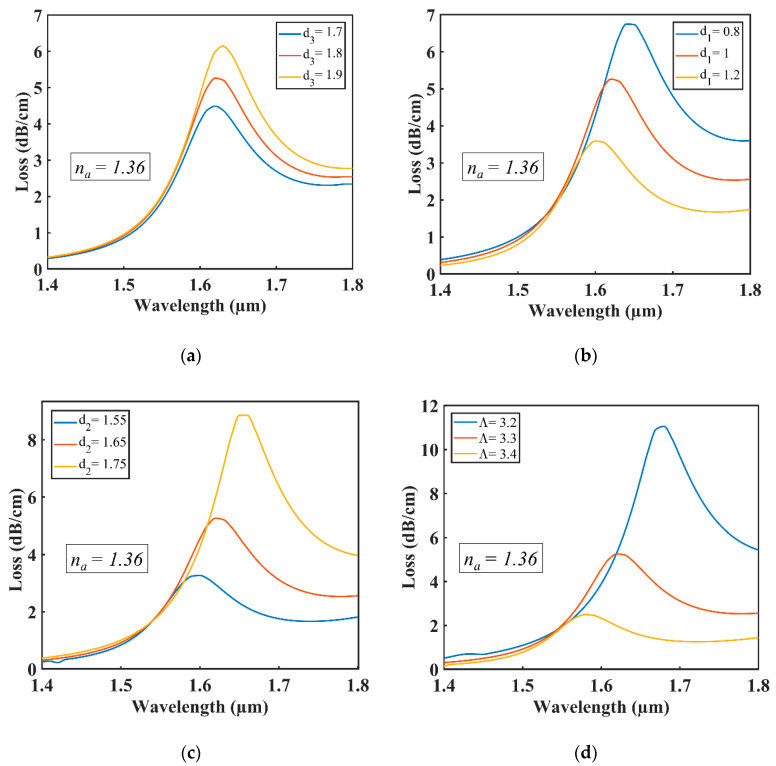
Loss curves with varying for (**a**) d_3_; (**b**) d_1_; (**c**) d_2_; (**d**) pitch Λ.

**Figure 6:**
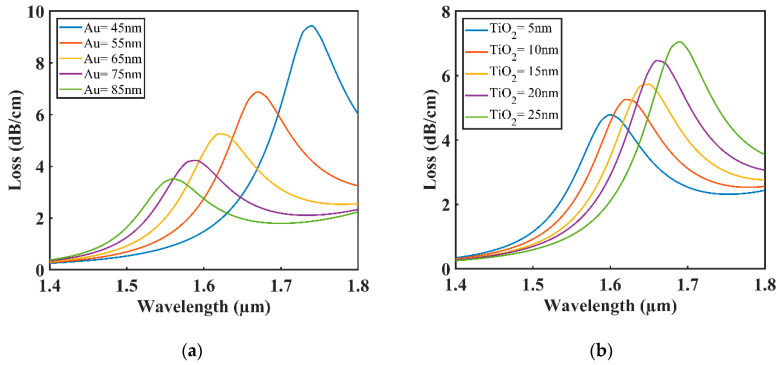
Loss curves with varying thickness for (**a**) Au (**b**) Titanium dioxide (TiO_2_).

At the resonant wavelength, the impact of particular parameters’ variation of the proposed sensor was analyzed for the RI of analyte *n_a_* = 1.36. At the same time, Gold was used as plasmonic material—see Figure 5 and Figure 6.

It is shown in Figure 5a that a minimal redshift with high confinement loss was observed as the established coupling strengthened between the core mode and the plasmonic mode when the air hole diameter of d_3_ increased from 1.7 to 1.9 µm. The redshifting occurs as the effective index of the fundamental mode and plasmonic mode changes with the changing of air hole diameter d_3_ and their phases matched at a higher wavelength point than the previous. Confinement loss increased due to the establishment of strong coupling between the core mode and plasmonic mode. Figure 5b shows that the confinement loss decreased with blueshift as the established coupling between the core mode and the plasmonic mode weakened when the air hole diameter of d_1_ increased from 0.8 to 1.2 µm. The blueshifting occurred as the effective index of the fundamental mode, and plasmonic mode changed with the changing of air hole diameter d_1_. Their phases matched at a lower wavelength point than the previous. Figure 5c shows a redshift followed by high confinement loss of resonant wavelength when the air hole d_2_ increased from 1.55 to 1.75 µm. Figure 5d also shows that changing the value of pitch from 3.2 to 3.4 µm resulted in a blueshift with low confinement loss. The optimized parameter values for d_3_, d_1_, d_2_, and pitch are 1.80, 1.00, 1.65, and 3.30 µm, respectively, as the highest sensitivities were achieved at these values for the proposed sensor.

The impact of changing the thickness of TiO_2_ and Au on loss curves is shown in Figure 6a,b. Increasing the Gold layer thickness increased the damping loss, which resulted in poor penetration in the evanescent field, which results in low confinement loss [28,29]. Significant blueshift was seen with low confinement loss when changing the thickness of Gold from 45 to 85 nm. When the effective RI of the fundamental mode and plasmonic mode changes, the phase-matching condition also changes [30,31]. Figure 6b demonstrates redshifting with high confinement loss in the resonant wavelength when the thickness of gold remained constant at 65 nm, and the thickness of the TiO_2_ layer was increased from 5 to 25 nm. Moreover, a significant contribution was made by the TiO_2_ layer to create a strong coupling between the core mode and plasmonic mode. The optimal values for Au and TiO_2_ were 65 and 10 nm, respectively, as the proposed sensor showed maximum performance at these values.

The amplitude sensitivity of the proposed sensor is defined by Equation (6) [19].

(6) $$AS\;[RIU^{-1}]=-\frac{1}{\alpha (\lambda ,n_{a})}\frac{\partial \alpha (\lambda ,n_{a})}{\partial n_{a}}$$

where, given the analyte’s loss for *RI*, *n_a_* is determined by *α*(*λ*, *n_a_*), the variance between two-loss spectra is specified by ∂*α*(*λ*, *n_a_*), and change in analyte *RI* is denoted by ∂*n_a_*.

**Figure 7:**
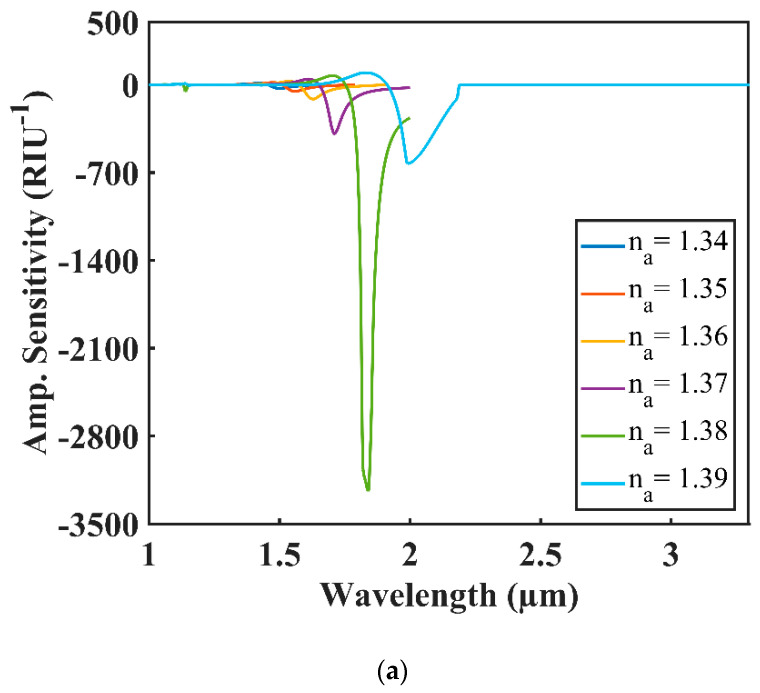

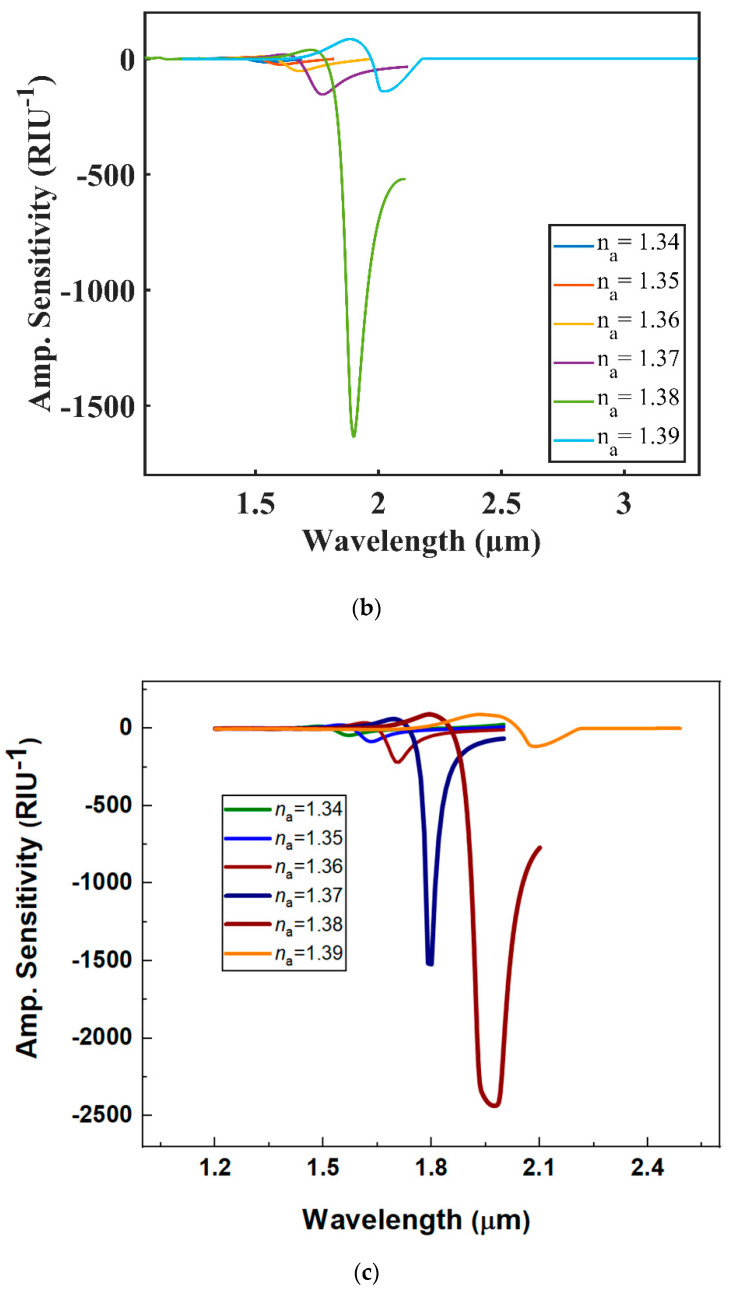
Amplitude sensitivity (AS) for the analyte RI varying from 1.34 to 1.39 of (**a**) Au; (**b**) Cu; (**c**) Ag.

The maximum amplitude sensitivity (AS) of 3239, 2452 and 1637 RIU^−1^ for *n_a_* = 1.38 was found for Au, Ag and Cu, respectively. The AS of the proposed sensor for changing analyte RI range from 1.34 to 1.39 for each plasmonic material is illustrated in Figure 7a–c.

**Figure 8:**
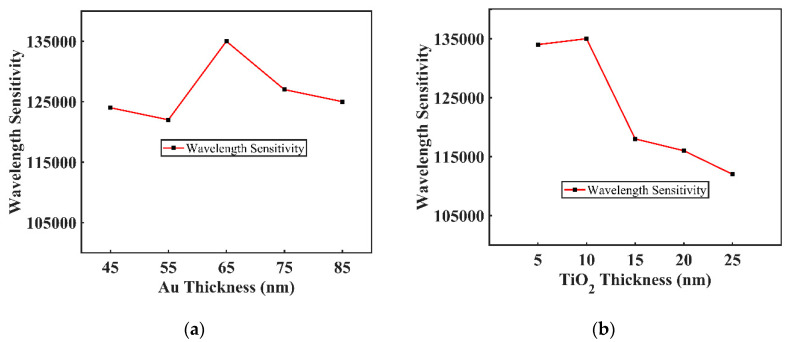
Wavelength sensitivity (WS) of the proposed sensor for varying (**a**) Gold thickness; (**b**) TiO_2_ thickness.

With the changing of Au and TiO_2_ thickness, the proposed sensor’s WS also varied accordingly which is illustrated in Figure 8a,b. After optimization, the maximum WS of 135,000 nm/RIU was achieved when defining a thickness of 10 nm for TiO_2_ and 65 nm for Au. Similarly, the maximum WS of 116,000 nm/RIU and 117,000 nm/RIU were also achieved for the same value of thickness for silver and copper, respectively. It is noted that the value of thickness for 10 nm TiO_2_ also remained the same in both cases.

The proposed Sensor’s performance for different bimetallic plasmonic material layers is illustrated in Table 1 and compared with other recently reported PCF-SPR sensors. As a result, it shows excellent performance compared to others. Furthermore, the lowest 8.62 × 10^−7^ RIU resolution was achieved when using plasmonic material silver, which shows that the proposed sensor can detect fractional micro-scale changes in analyte [28].

## 4. Conclusions

A highly sensitivity SPR sensor with a dual-core configuration for low RI detection was studied numerically and compared to three different plasmonic materials (Au, Ag, and Cu). The maximum WS of 135,000, 116,000, and 117,000 nm/RIU and resolution of 7.41 × 10^−7^, 8.62 × 10^−7^, and 8.54 × 10^−7^ RIU was achieved for the plasmonic material Au, Ag and Cu, respectively. The maximum AS of 3239 (Au), 2452 (Ag) and 1637 RIU^−1^ (Cu) was obtained. The proposed sensor’s fabrication difficulties were minimized as the sensing medium was outside, which makes this sensor more feasible for practical fabrication. The proposed sensor achieved a FOM of 3375, 2320 and 3900 for Au, Ag, and Cu, respectively, which are very high compared to other sensors. These qualities make this sensor more appealing for use in identifying analytes that have a lower RI in bio-sensing, organic chemical detection, water quality testing and so on.

## Figures and Tables

**Table 1:** Performance comparison of the proposed sensor with other recent reported photonic crystal fiber (PCF) sensors.

Ref.		RI Range	Max AS	Max WS	Max FOM	Lowest.R
[32]	Gold	1.33–1.43	1415	62,000	1140	1.6 × 10^−6^
[16]	Gold	1.22–1.37	1872	51,000	566	1.96 × 10^−6^
[19]	Gold	1.33–1.43	1086	46,000	-	2.2 × 10^−6^
[21]	Gold	1.32–1.40	-	31,000	-	3.31 × 10^−5^
[33]	Indium Tin Oxide	-	74	17,000	-	5.8 × 10^−6^
[34]	Niobium	1.36–1.41	1560	8000	266	1.25 × 10^−5^
Proposed	Gold	1.29–1.39	3239	135,000	3375	7.41 × 10^−7^
	Silver		2452	116,000	2320	8.62 × 10^−7^
	Copper		1637	117,000	3900	8.54 × 10^−7^
